# Minimally invasive separation surgery for the treatment of spinal metastases with small incision and freehand pedicle screw fixation: the surgical learning curve

**DOI:** 10.1186/s12891-022-05191-2

**Published:** 2022-03-22

**Authors:** Jiaming Lin, Xiaojun Zhu, Qinglian Tang, Jinchang Lu, Huaiyuan Xu, Guohui Song, Chuangzhong Deng, Hao Wu, Yufeng Huang, Anfei Huang, Yanyang Xu, Hongmin Chen, Jin Wang

**Affiliations:** Department of Musculoskeletal Oncology, Sun Yat-Sen University Cancer Center, State Key Laboratory of Oncology in South China, Collaborative Innovation Center for Cancer Medicine, 651 Dongfeng East Road, Guangzhou, 510060 Guangdong China

**Keywords:** Bone metastases, Minimally invasive separation surgery (MISS), Malignant tumor, Myelopathy, Surgical learning curve, Spinal metastases

## Abstract

**Background:**

Minimally invasive separation surgery (MISS) is a safe and effective surgical technique, the current optimal treatment for spinal metastases. However, the learning curve for this technique has not been analyzed. This study aimed to define and analyze the surgical learning curve of MISS for the treatment of spinal metastases with small incision and freehand pedicle screw fixation.

**Methods:**

A continuous series of 62 patients with spinal metastases who underwent MISS were included. Each patient's operative data were accurately counted. The improvement of the patients' neurological function was followed up after surgery to evaluate the surgical treatment effect. Logarithmic curve-fit regression was used to analyze the surgical learning curve of MISS. The number of cases needed to achieve proficiency was analyzed. Based on this cut-off point, this series of cases was divided into the early phase and later phase groups. The influence of the time sequence of MISS on surgical data and surgical efficacy was analyzed.

**Results:**

The operative time decreased gradually with the number of surgical cases increasing and stabilized after the 20th patient. There was no statistical difference in demographic characteristics and preoperative characteristics between the two groups. The mean operative time in the later phase group was about 39 min shorter than that in the early phase group (mean 227.95 vs. 189.02 min, *P* = 0.027). However, it did not affect other operative data or the surgical treatment effect.

**Conclusion:**

The learning curve of MISS for spinal metastases is not steep. With the increase of surgeons' experience, the operative time drops rapidly and stabilizes within a certain range. MISS can be safely and effectively performed at the beginning of a surgeon's caree.

## Background

About 70% of patients with cancer will develop spine metastases, and 10% will suffer from metastatic spinal cord compression causing neurological symptoms, pain, and reduction in quality of life (QOL) [1, 2]. Thus, the main goal of treating spinal metastasis is to improve QOL as much as possible by preserving neurological function [3] and relieving pain [4]. Radical surgery, such as total en-bloc spondylectomy, is not suitable for most patients with multiple spinal lesions regarding the perioperative complications and limited life expectancy. Instead, palliative surgery, such as separation surgery, is usually recommended [5]. Researches at different cancer centers are increasingly recommending MISS as the current optimal treatment for spinal metastases [6–12]. Compared with traditional open surgery (TOS), MISS has the advantages of less trauma, less blood loss, lower incidence of complications, and shorter hospital stay without compromising operative duration and functional outcomes [13].

Trying a new surgical technique without knowing this technique's learning curve may lead to repetitive or unnecessary mistakes. More importantly, surgeons need to be aware of the risks of performing the surgical technique early in their career and predict when proficiency is expected to be achieved. Several studies have analyzed the learning curve of different minimally invasive surgical techniques used to treat spinal diseases, contributing to the understanding and evaluation of these new techniques by surgeons and the popularization of these new techniques [14–17]. However, no studies have described the learning curve of MISS. To our best knowledge, no studies have analyzed the course of learning the technique by a single surgeon in a series of cases. Therefore, this study attempted to define and analyze the learning curve of using minimally invasive surgical techniques for separation surgery and to evaluate the usefulness of this technique.

## Methods

### Data collection

From December 2018 to September 2020, a continuous series of patients with spinal metastases who underwent MISS in our center were collected, approved by the Sun Yat-sen University Cancer Center Ethics Committee. Indications of surgery for patients with spinal metastasis were the presence of progressive paralysis due to spinal cord compression or intolerable back pain as a result of the instability of pathologic fracture [6]. Patient survival was assessed using TOMITA and TAKUHASHI revised scoring systems, with all patients expected to survive more than 3 months after surgery. Inclusion criteria were patients who met the indications of separation surgery, underwent single-level MISS (decompression in one segment), and underwent surgery at the surgical site for the first time. Patients who underwent double-level or multi-level MISS or only had lamina involvement were excluded.

A total of 62 patients were eventually included in the study, with a time span of about 22 months. All patients have signed the documentation of operation consent. These cases were treated in a single treatment group, using the same surgical technique, and performed by the same junior attending and his surgical team members. The doctor had not performed this kind of surgery before the first patient but had been formally trained in the surgical technique. The series of cases were sorted and numbered in chronological order by the date of operation.

Patients’ Demographic and Basic Clinical Data such as age, gender, type of primary tumor, number of spinal metastases, surgical site, and co-morbidities in this series were collected. Patients included underwent a standard preoperative evaluation, and preoperative embolization, epidural spinal cord compression (ESCC), spinal cord injury Frankel grades, and Karnofsky performance status (KPS) were carefully evaluated. Each patient's operative data were also accurately counted, including the operative time, intraoperative blood loss, postoperative drainage volume, the retention time of drainage tube, postoperative hospital stay, perioperative complications (Including wound issues, infection, hardware failure, hematoma, dural rupture, durotomy, cerebrospinal fluid leaks, tumor recurrence), and other variables. The improvement of the patients' neurological function was followed up four weeks after surgery using Frankel grades to evaluate the surgical treatment effect further.

### Surgical technique

**Fig. 1:**
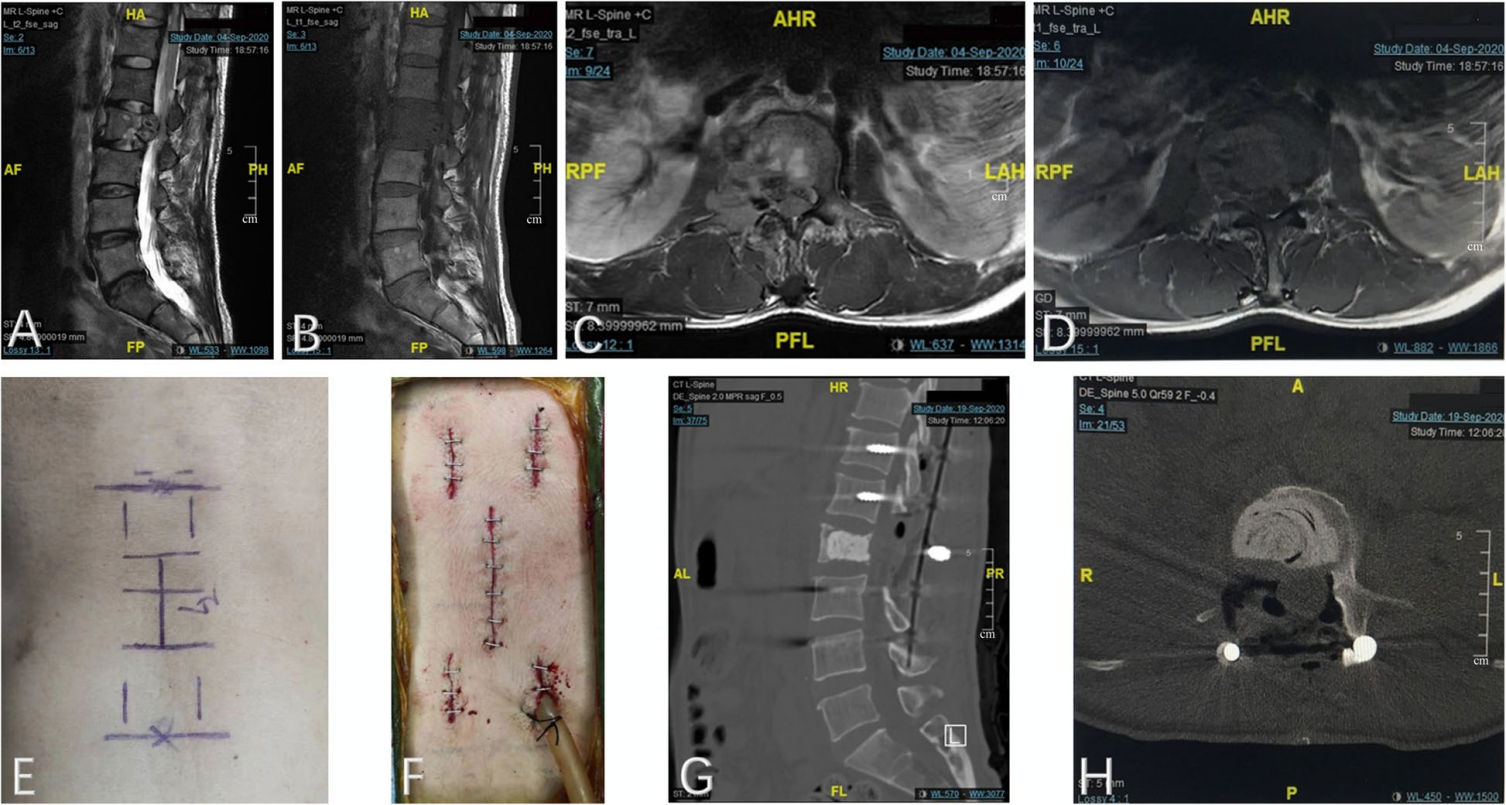
A representative case: a 52-year-old man with L2 hepatic carcinoma metastasis. **A**-**D** The T2and T1-weighted sagittal (**A** and **B**) and axial (**C **and **D**) MRI images showed a destructive lesion involving L2 with high-grade ESCC (Grade 2). **E**–**F** Photographs showing the 5–6 cm incision in the midline for tumor debulking and spinal cord decompression (**E**). Another four small incisions (1.5-2 cm) were made for pedicle screws and rods implantation (**F**). **G**-**H** The sagittal (**G**) and axial (**H**) CT images demonstrated pedicle screws and rods position and PMMA after minimally invasive separation surgery (MISS)

The procedure and characteristics of MISS for the treatment of spinal metastases with a small incision and freehand pedicle screw fixation are as follows. The separation surgery was performed in a posterolateral approach that allows for stabilization and circumferential epidural decompression to create at least 2- to 3-mm epidural margin for ablative SBRT within the constraints of spinal cord tolerance [18]. The "C" arm X-ray machine located the position of the affected vertebra and the position of the pedicle of its upper and lower two segments, and marked them as the corresponding incision. The pedicle screws were implanted with a 1- to 2-cm small incision through the Wiltse paravertebral muscle space approach under direct vision, followed by circumspinal decompression along bilateral sides through a 5- to 6-cm skin midline incision. Pedicle screws were placed a minimum of 2 levels above and below the decompression level to support the vertebral lesion. Finally, the connecting rods were inserted subcutaneously by the incision above. For osteolytic and mixed lesions, PMMA was applied to fill the vertebral lesion site through a bone cement push rod to support the spinal stability further. After decompression was completed, tumor samples were taken for histopathological analysis. A non-suction drain was placed at the end of the procedure. For tumors of the thoracic vertebrae, a neuroelectrophysiological detection system (Protektor32, Natus Medical Incorprated DBA Excel-Tech Lid, Canada.) was used for intraoperative detection. Figure 1 shows a representative case.

### Statistical analysis

Logarithmic curve-fit regression was used to analyze the surgical learning curve of MISS in this series of cases. The method has been reported in previous studies [14, 15, 17]. The number of cases needed to achieve proficiency was analyzed. Based on this cut-off point, this series of cases was divided into the early phase and later phase groups (technical steady-state group). Statistical analyses were performed using SPSS software, version 16.0 (SPSS Inc., Chicago, IL, US). The independent sample t-test and the χ2 or Fisher's exact test were performed for continuous and categorical variables. Firstly, the two groups of patients were analyzed to see if there were differences in demographic characteristics and preoperative characteristics. The influence of the time sequence of MISS on surgical data and surgical efficacy was further analyzed. Significance was set at *P* value of less than 0.05.

## Results

The learning curve of MISS as shown by operative time.

**Fig. 2:**
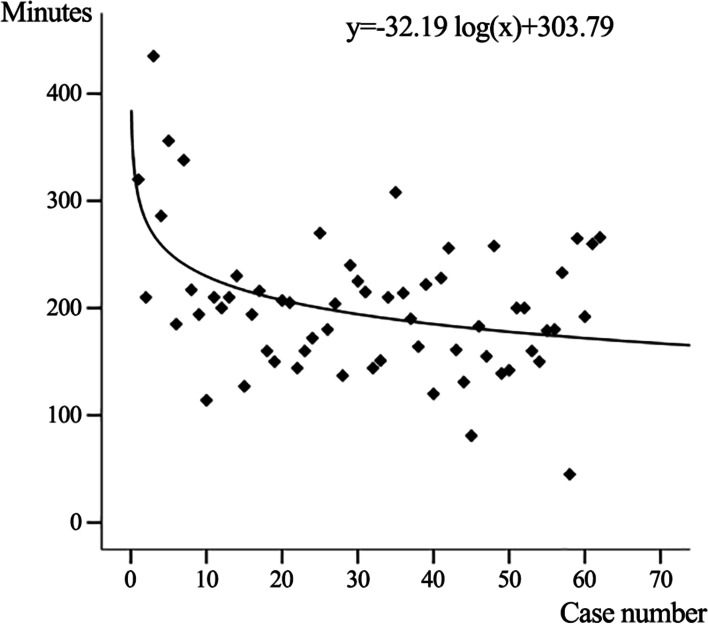
The learning curve of minimally invasive separation surgery (MISS) as shown by operative time

As the number of surgical procedures performed by the surgeon increased, the operative time of the minimally invasive separation procedure gradually decreased and stabilized after the 20th patient, as indicated by the equation (y = -32.19 log(x) + 303.79, *P* < 0.001, *R*^*2*^ = 0.197) (Fig. 2).

### Patients’ demographic and basic clinical data

**Fig. 3:**
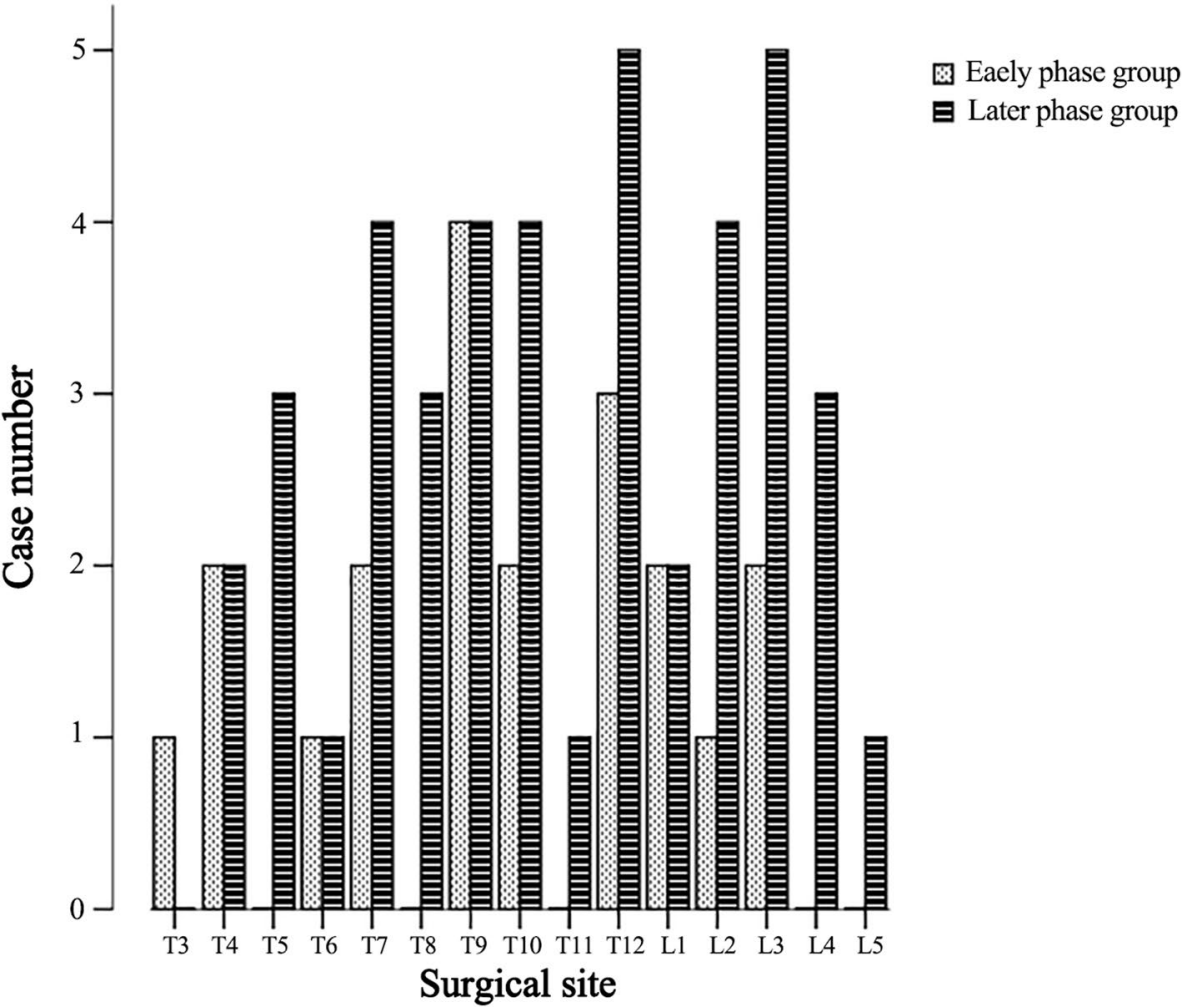
The distribution of the surgical site in the vertebral body

The 20^th^ patient was taken as the cut-off point, and the 20^th^ and before patients were classified as the early phase group, while those after the 20^th^ patient were classified as the later phase group. There were no significant differences in age (*P* = 0.906) or gender (*P* = 0.544) between the two groups (Table 1). Except for the 50^th^ patient (1 patient in the later phase group), all the other patients had multiple spinal metastases, and there was no statistical difference between the two groups (*P* = 0.677) (Table 1). The surgical site's distribution in the vertebral body is shown in Fig. 3. Statistical analysis showed no difference in the distribution of the surgical site in the thoracic and lumbar vertebrae (*P* = 0.294) (Table 1). As for co-morbidities such as coagulation dysfunction and diabetes, these two factors did not differ statistically between the two groups (Table 1). The distribution of primary tumor types was shown in Table 2, and the top three were breast cancer (13 cases,21.0%), lung cancer (12 cases,19.4%), and renal cancer (7 cases,11.3%). There was no statistical difference in the distribution of primary tumor types between the two groups (*P* = 0.318) (Table 2).

**Table 1:** Patients’ Demographic and Basic Clinical Data

	**Early phase group (*n* = 20)**	**Later phase group (*n* = 42)**	***P* value**
Age(mean ± SD)	54.35 ± 10.48	53.98 ± 12.02	0.906
Gender (%)			0.544
Female	11(55%)	24(57.1%)	
Male	9(45%)	18(42.9%)	
Number of spinal metastases (%)			0.677
multiple	20(100%)	41(97.6%)	
single	0	1(2.4%)	
Surgical site (%)			0.294
Thoracic	15(75%)	27(64.3%)	
lumbar	5(25%)	15(35.7%)	
Coagulation dysfunction			
Normal	18(90%)	42(100%)	0.189
Abnormal	2(10%)	0	
Diabetes			
Normal	20(100%)	39(92.9%)	0.554
Abnormal	0	3(7.1%)	

*SD* standard deviation. The criteria for abnormal coagulation dysfunction were: prothrombin time (PT) has been increased by 3 s or activated partial thromboplastin time (APTT) increased by 10 s

**Table 2:** The Distribution of Primary Tumor Types

Primary tumor	Early phase group (%)	Later phase group (%)	*P* value
Breast	2(10%)	11(26.2%)	
Lung	3(15%)	9(21.4%)	
Kidney	5(25%)	2(4.8%)	
Liver	1(5%)	4(9.5%)	
Thyroid	2(10%)	1(2.4%)	
Myeloma	2(10%)	3(7.1%)	
Colorectal	0	1(2.4%)	0.318
Unknow	1(5%)	3(7.1%)	
Prostate	1(5%)	0	
Nasopharynx	1(5%)	2(4.8%)	
Uterus	1(5%)	1(2.4%)	
Other	1(5%)	5(11.9%)	
Total	20(100%)	42(100%)	

### Preoperative evaluation of spinal metastases

A comparative analysis was made of the preoperative evaluation of the two groups of cases. One patient in each group had undergone preoperative embolization, and the primary tumor type in both cases was renal carcinoma. There was no statistical difference in preoperative embolization between the two groups (*P* = 0.545) (Table 3). Besides, there were no statistically significant differences between the two groups in preoperative Frankel grades (*P* = 0.270), ESCC (*P* = 0.651), and KPS (*P* = 0.255) (Table 3).

**Table 3:** Preoperative Evaluation of Spinal Metastases

	Early phase group (*n* = 20)	Later phase group (*n* = 42)	*P* value
Preoperative embolization (%)			0.545
yes	1(5%)	1(2.4%)	
no	19(95%)	41(97.6%)	
Preoperative Frankel grades (%)			0.270
A	2(10%)	1(2.4%)	
B	0	0	
C	2(10%)	12(28.6%)	
D	10(50%)	18(42.9%)	
E	6(30%)	11(26.2%)	
ESCC (%)			0.651
1A	0	1(2.4%)	
1B	2(10%)	5(11.9%)	
1C	2(10%)	5(11.9%)	
2	10(50%)	13(31.0%)	
3	6(30%)	18(42.9%)	
KPS (%)			0.255
≥ 60	13(65%)	22(52.4%)	
< 60	7(35%)	20(47.6%)	

*ESCC* epidural spinal cord compression; *KPS* Karnofsky performance status

### Surgical data of MISS

In the analysis of operative data, it was found that the mean operative time of the later phase group was significantly reduced compared with the early phase group, with a mean reduction of about 39 min, and the difference between the two groups was statistically significant (*P* = 0.027) (Table 4). On the other hand, although the operative time of the later phase group was significantly shortened, it did not affect the intraoperative blood loss (*P* = 0.533), the incidence of massive blood loss (> 1000 ml) (*P* = 0.505), retention time of drainage tube (*P* = 0.385), the length of postoperative hospital stay (*P* = 0.622) and the incidence of perioperative complications (*P* = 0.696), and there was no statistical difference in the above variables between the two groups (Table 4). Among the patients with intraoperative massive blood loss, there were four patients in the early phase group, including two patients with spinal metastases from renal carcinoma and the remaining two patients with spinal metastases from breast cancer and thyroid cancer, respectively. In the later phase group, there were 10 cases with intraoperative massive blood loss, of which the primary tumor type was breast cancer in 5 cases, liver cancer in 2 cases, myeloma in 2 cases, and thyroid cancer in 1 case. In terms of postoperative drainage volume, there was no significant difference in postoperative drainage volume between the two groups after the extreme value was removed (*P* = 0.054) (Table 4). As for perioperative complications, one patient in the early phase group developed dural rupture. Two patients in the later phase group developed an epidural hematoma. There is no postoperative infection that occurred in either group.

**Table 4:** Surgical Data of Minimally Invasive Separation Surgery (MISS)

	Early phase group (*n* = 20)	Later phase group (*n* = 42)	*P* value
Operative time (min) (mean ± SD)	227.95 ± 80.69	189.02 ± 53.52	0.027
Intraoperative blood loss (ml) (mean ± SD)	686.50 ± 631.07	792.86 ± 532.89	0.533
Massive blood loss (> 1000 ml) (%)	4(20%)	10(23.8%)	0.505
Postoperative drainage volume (ml) (mean ± SD)	337.61 ± 253.51	471.88 ± 235.01	0.054
Retention time of drainage tube (min) (mean ± SD)	4.44 ± 1.67	4.88 ± 1.51	0.385
Postoperative hospital stay (day) (mean ± SD)	5.25 ± 2.27	4.93 ± 2.44	0.622
Perioperative complications (%)	1(5%)	2(4.8%)	0.696

*SD* standard deviation

### The improvement of neurological function after operation

The improvement of neurological function after the operation was statistically analyzed. There was no significant difference in the improvement of neurological functional status (*P* = 0.600) and Frankel grades improvement (*P* = 0.827) between the two groups (Table 5). Neurological function improved in 6 of 20 patients (30%) in the early phase group and 13 of 42 patients (31.0%) in the later phase group. Postoperative neurologic function was stable in 14 (70%) and 27 (64.3%) patients in the early phase and later phase groups respectively. Five patients (25%) in the early phase group had grade 1 improvement, and one (5%) had grade 2 improvement. In the later phase group, 12 patients (28.6%) had grade 1 improvement, and one (2.4%) had grade 2 improvement. In general, the surgical treatment effect of the two groups was satisfactory.

**Table 5:** The Improvement of Neurological Function after Operation

	Early phase group (*n* = 20)	Later phase group (*n* = 42)	*P* value
Improvement of neurological functional status			0.600
improve	6(30%)	13(31.0%)	
stable	14(70%)	27(64.3%)	
worse	0	2(4.8%)	
Frankel grades improvement			0.827
grade 0	14(70%)	27(64.3%)	
grade 1	5(25%)	12(28.6%)	
grade 2	1(5%)	1(2.4%)	

## Discussion

In clinical practice, the learning curve associated with surgery is an essential consideration when advising patients about the possible treatment options and their expected efficacy. Besides, after the safety and effectiveness of a new surgical technique have been verified, this technique's promotion and popularization also need to consider the learning curve. MISS is a safe and effective surgical technique, the current optimal treatment for spinal metastases [6–12]. However, the learning curve for this technique has not been analyzed. This study described the learning curve of MISS, and analyzed and compared preoperative, intraoperative and postoperative objective factors related to this surgical technique's learning curve.

In this series of patients with spinal metastases, the operative time-based learning curve of MISS showed that the operative time decreased gradually with the number of surgical cases increasing and became stabilized after the 20th patient. Based on this, this series of cases were divided into the early phase group and the later phase group. There was no statistical difference in demographic characteristics and preoperative characteristics between the two groups. Under this premise, we analyzed and compared the surgical data and clinical efficacy of the two groups of patients. The mean operative time was significantly shorter in the later phase group than that in the early group, about 39 min shorter on average. It demonstrated that after the appropriate number of surgical cases, the surgeon can quickly reach the technical steady period, and the operative time is stable within a certain range. Crucially, shorter operative time in the later phase group did not affect operative data. There were no statistically significant differences between the two groups in intraoperative blood loss, the incidence of massive blood loss, postoperative drainage volume, the retention time of drainage tube, postoperative hospital stay, and incidence of perioperative complications. Moreover, it is worth noting that both groups of patients obtained good postoperative outcomes, and there was no statistical difference in surgical treatment effect between the two groups. In both groups, neurological function improved to varying degrees or remained stable in most patients.

With the progress of comprehensive treatment of tumors, the number of patients with spinal metastases is increasing. Therefore, it is necessary to promote and popularize the MISS technique. Although many cancer centers have developed this technique successively, many surgeons are still concerned that the small incision of MISS will increase the difficulty of the operation and that it is difficult to stop intraoperative tumor bleeding through a small incision, etc. These worries may be caused by the fact that the learning curve of MISS was not clearly defined and analyzed before this study. By defining and analyzing the learning curve of MISS, this study shows that this surgical technique's learning curve is not steep, and surgeons could reach the technical proficiency period after receiving formal training and an appropriate number of surgical cases. Although the initial operative time is longer than that in the latter stage, the surgical treatment effect is comparable to that of the latter cases, and the effect is satisfactory. Therefore, we believe that after the training of minimally invasive separation procedures, surgeons can gradually carry out this surgical technique so that more patients with spinal metastases can benefit from this minimally invasive technique.

According to Selafani's systematic review, for most minimally invasive spinal surgery techniques, continuous completion of 20–30 procedures can overcome the learning curve marked by operative time and incidence of complications [19]. Therefore, a beginner must pay attention to the prevention of complications when developing new technology and find some regular experience in literature study and attending academic conferences to go through the learning curve smoothly. For MISS, our recommendations to surgeons who are performing this technique in the early stages are as follows. Firstly, before carrying out this operation, it is necessary to have a specific basis in spinal surgery and bone oncology and to receive surgical skill training from a professional department. Otherwise, the probability of spinal cord or nerve root injury during the operation may increase. Secondly, for metastatic spinal lesions with a rich blood supply (such as kidney cancer, thyroid cancer, liver cancer, etc.) [20, 21], preoperative embolization can be considered to reduce intraoperative bleeding. Thirdly, when the spinal cord or nerve root decompression is performed, it is better to carry out electrophysiological nerve monitoring. After returning to the ward after surgery, it is necessary to ensure that the drainage tube is patency and closely observe the changes in nerve function. If problems are found, timely treatment should be carried out.

The study is not without limitations. Firstly, this was a retrospective study, not a prospective one, making it difficult to avoid the cofounder bias, although the early and late group cohorts seemed to match well. Secondly, long-term QOL was not assessed due to the short follow-up time.

## Conclusion

The learning curve of MISS for spinal metastases is not steep. With the increase of surgeons' experience, the operative time drops rapidly and stabilizes within a certain range. MISS can be safely and effectively performed at the beginning of a surgeon's career. This conclusion may be useful for young surgeons to choose between MISS and TOS to treat spinal metastases. It is also evidence of the high feasibility of the popularization of this technique.

## Data Availability

All data analyzed in this study has been provided in the manuscript.
